# Complete Remission of Anaplastic Thyroid Carcinoma after Concomitant Treatment with Docetaxel and Radiotherapy

**DOI:** 10.1155/2015/726085

**Published:** 2015-02-18

**Authors:** Ichiro Abe, Satoko Karasaki, Yayoi Matsuda, Shohei Sakamoto, Torahiko Nakashima, Hidetaka Yamamoto, Hisaya Kawate, Keizo Ohnaka, Hisashi Nakashima, Kunihisa Kobayashi, Yoshinao Oda, Masatoshi Nomura, Ryoichi Takayanagi

**Affiliations:** ^1^Department of Medicine and Bioregulatory Science, Graduate School of Medical Science, Kyushu University, Maidashi 3-1-1, Higashi Ward, Fukuoka 812-8582, Japan; ^2^Department of Endocrinology and Diabetes Mellitus, Fukuoka University Chikushi Hospital, 1-1-1 Zokumyoin, Chikushino, Fukuoka 818-8502, Japan; ^3^Department of Otorhinolaryngology, Graduate School of Medical Science, Kyushu University, Maidashi 3-1-1, Higashi Ward, Fukuoka 812-8582, Japan; ^4^Department of Anatomic Pathology, Graduate School of Medical Science, Kyushu University, Maidashi 3-1-1, Higashi Ward, Fukuoka 812-8582, Japan; ^5^Department of Internal Medicine, Haradoi Hospital, Aoba 6-40-8, Higashi Ward, Fukuoka 813-8588, Japan

## Abstract

Anaplastic thyroid carcinoma (ATC) although rare is the most lethal form of thyroid cancer. The mortality rate for ATC is very high, with a median survival time of only 5 months; the survival rate at 1 year after diagnosis is <20%. Management of ATC is extremely difficult and rife with uncertainties. Herein, we describe a 75-year-old woman who presented with ATC and was successfully treated using concomitant treatment with docetaxel and high-dose radiotherapy. This case appears to be the first to have been reported in the literature involving complete remission of ATC confirmed by autopsy, suggesting the therapeutic potential of this combination.

## 1. Introduction

Anaplastic thyroid carcinoma (ATC) is a rare carcinoma that accounts for 2% of all thyroid cancers [1] and has a slightly higher rate of occurrence in women; its incidence peaks in the sixth and seventh decades of life. ATC is one of the most aggressive cancers with patients having a median survival time of only 5 months; <20% of patients are alive at 1 year after diagnosis. The disease is locally advanced with infiltration of surrounding organs and blood vessels. Furthermore, 50% of patients have distant metastasis at diagnosis [2]. All patients are classified as stage IV, with the primary lesion restricted to the thyroid gland in stage IVA disease; locoregional lymph nodes may exist in stages IVA/IVB and stage IVC is defined by distant metastasis, according to the American Joint Commission on Cancer TNM staging system. It has long been thought that the most important factors associated with longer survival are completeness of surgical resection and high-dose external beam radiotherapy. Because progression of ATC is extremely rapid and no effective systemic chemotherapy has been established, its management is quite difficult and rife with uncertainties. The problems include the following: (1) should adjuvant radiotherapy and/or chemotherapy be used in the treatment of stage IVA disease; (2) which chemotherapy regimens should be recommended for stage IVB unresectable disease; (3) which systemic therapies should be considered for stage IVC patients; and (4) which clinical factors should be used to direct an appropriate therapeutic approach to the individual patient or help patients in their decision making regarding the pursuit of aggressive therapy or palliative/hospice care. All of these problems might be resolved when an effective treatment strategy including systemic chemotherapy has been established. Recently, high-dose radiotherapy plus combination chemotherapy has been shown to improve long-term survival in ATC patients. In particular, the combination of radiotherapy and concomitant docetaxel has been reported to be highly effective [3]. Here, we report a case of complete remission of ATC at stage IVB after treatment using concomitant docetaxel and radiotherapy. In addition, complete remission was pathologically confirmed at autopsy, suggesting the therapeutic potential of this treatment combination.

## 2. Case Presentation

**Figure 1:**
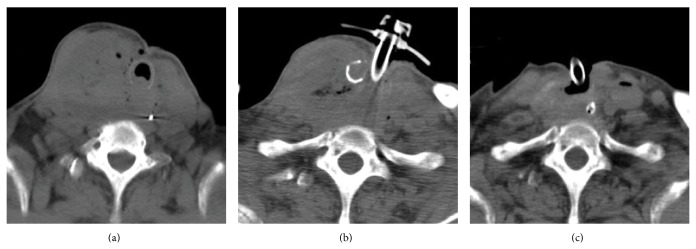
ATC in a 75-year-old woman. The cervical mass was inseparable from the strap muscles, tracheal wall, and carotid sheath. (a) Computed tomography scan showing a huge mass in the right thyroid robe on admission, (b) after surgical resection of a part of tumor and tracheotomy, and (c) after radiotherapy and seven cycles of chemotherapy.

**Figure 2:**
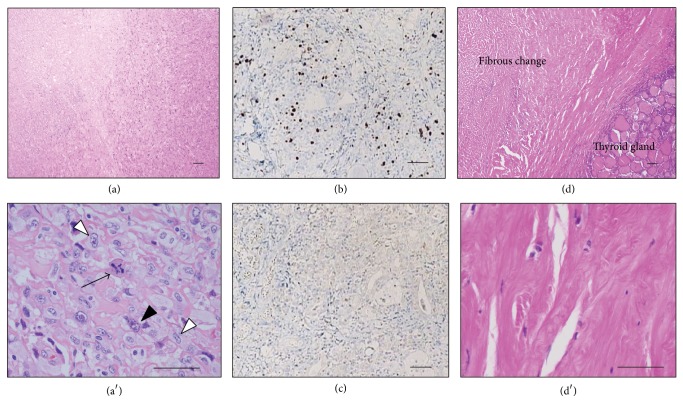
Pathological analysis of anaplastic thyroid cancer. (a, a′) Hematoxylin and eosin (HE) stained tumor section. High magnification is shown in the lower panel (a′). The tumor cells were composed of polygonal cells (closed arrowhead), spindle cells (open arrowheads), and multinucleated cells (arrow) with necrosis. (b) The Ki-67 labeling index was 50%. (c) The tumor cells were negative for p53. (d, d′) HE stained section of the thyroid gland after autopsy. It should be noted that the tumor is completely substituted by fibrotic tissue. Scale bars = 50 *μ*m.

A 75-year-old woman presented at the nearby hospital with the complaints of neck swelling and respiratory discomfort. She had been diagnosed with type 2 diabetes mellitus 20 years previously and had been treated exclusively with diet therapy. The risk factors for ATC remain to be clarified [4]. She did not have any other past history such as thyroid tumor and special medical history. A right thyroid mass was detected using sonogram. Because a malignant tumor was suspected, the patient was referred to our hospital for further examination and treatment. A sonogram confirmed the presence of a very large tumor (46 × 44 × 78 mm) with homogenous appearance in the right thyroid lobe. The left thyroid lobe was dislocated to the opposite side. Abnormal cervical lymph nodes could not be detected by a sonogram. A diagnosis of undifferentiated carcinoma was made using fine needle aspiration cytology, and ATC was highly suspected. On physical examination, the patient presented with swelling of the neck and her peripheral capillary oxygen saturation (SPO_2_) level was 93% (in room air). Her blood pressure was 124/78 mmHg and her body temperature was 37.1°C. The patient had a regular pulse rate, which was 100/min. Laboratory tests revealed an elevated white blood cell count of 12,110/mm^2^ with 85.2% neutrophils and a C-reactive protein level of 13.98 mg/dL. The hemoglobin level was 10.0 g/dL and the platelet count was 541,000/*μ*L. On biochemical investigation, the blood glucose level was found to be 276 mg/dL and the HgbA1c level was 10.0%. On thyroid function tests, TSH was 3.05 *μ*U/mL (reference range: 0.30–4.30 *μ*U/mL) and free T4 was 1.23 ng/dL (reference range: 0.90–1.70 ng/dL). However, she had elevated levels of anti-thyroid antibodies, an anti-thyroglobulin antibody: 407.6 U/mL (reference range: <28 U/mL), and an anti-microsomal antibody: 1,732 U/mL (reference range: <16 U/mL). Thyroglobulin was 4.0 ng/mL (reference range: <32.7 ng/mL). An irregularly shaped very large mass in the right thyroid together with tracheal compression was detected using a computed tomography (CT) scan as shown in Figure 1(a). A further examination using a whole-body CT revealed that the thyroid tumor was the only tumor present. To obtain an appropriate diagnosis of this thyroid tumor and to prevent asphyxiation, surgical resection of a part of tumor and tracheotomy were conducted (Figure 1(b)). Histological analysis of the tumor using hematoxylin and eosin staining revealed that the tumor was composed of polygonal cells, spindle cells, and multinucleated cells; necrosis was also present (Figures 2(a) and 2(a′)). Using immunohistochemistry, the tumor cells were negative for TTF1 (data not shown). An extremely high number of the cells were found to be positive for Ki-67, indicating a highly proliferative tumor (Figure 2(b)). Although the tumor cells were negative for p53 (Figure 2(c)), known to be positive for 60% of ATCs [5], based on these pathological findings and the clinical manifestation of the thyroid tumor, ATC at stage IVB was diagnosed.

After making a diagnosis of stage IVB ATC, combined therapy with docetaxel and radiation was started. The patient received external beam radiotherapy at a total dose of 46 Gy delivered in 2 Gy daily fractions. Docetaxel was administered intravenously at a dose of 40 mg/m^2^ every 4 weeks; administration was initiated within the 1st week of radiotherapy. During the combined therapy, no severe adverse reactions developed, with the exception of mild radiation dermatitis. After the 3rd cycle of chemotherapy, a slight degree of tumor shrinkage of the thyroid tumor was observed on CT. The patient was discharged after the 4th cycle of chemotherapy. A whole-body PET-CT scan after 4th chemotherapy showed the right neck mass to be hypermetabolic with no metastasis. The patient continued chemotherapy with the same regimen every 8 weeks for >1 year (total 7 cycles) and further shrinkage of the thyroid tumor was observed; finally, the thyroid tumor could barely be detected on CT (Figure 1(c)). Unfortunately, the patient died suddenly 13 months after she referred to our hospital and 1 month after the seventh chemotherapy, with no worsening of the disease. Autopsy was carried out and the cause of death was identified as suffocation from sputum. Despite an extensive histopathological examination, there was no evidence of distance metastasis or malignant tissue in the thyroid gland; the tumor had been totally substituted with fibrotic tissue (Figures 2(d) and 2(d′)) indicating that the patient's ATC was in a complete remission.

## 3. Discussion

Because the progression of ATC is extremely rapid and aggressive, most patients initially present with a rapidly enlarging tumor in the neck, often causing progressive symptoms such as dyspnea. The disease is locally advanced with infiltration of the surrounding organs [2]. In the present case, the patient initially presented with a very large locally invasive tumor in the neck, which was diagnosed as stage IVB disease. Therefore, total resection of ATC could not be performed and systemic chemotherapy was concomitantly administered with radiotherapy to the localized lesion. An optimal treatment for ATC has not yet been established [6, 7]. American Thyroid Association guidelines for patients with stage IVA/IVB ATC recommended a multimodal approach including surgery, radiation, and systemic chemotherapy [8]. The ATC Research Consortium of Japan (ATCCJ) also reported that multimodal aggressive approach was effective to selected patients with ATC [9]. Cytotoxic drugs alone for advanced ATC are poorly effective. On the other hand, targeted biological agents including antiangiogenic agents and tyrosine kinase inhibitors might represent a viable therapeutic option. Axitinib [10], combretastatin A4 [11], sorafenib [12], and imatinib [13] have been tested in small clinical trials of ATC, with a promising disease control rate ranging from 33% to 75%. However, these findings have been derived from limited number of the patients. ATC is rare and thus it is difficult to advance evidence level of the diverse treatment of this deadly disease. Recently, Troch et al. reported that five ATC patients, who received radiotherapy at total doses of 50–60 Gy concomitantly with docetaxel administered intravenously at an absolute dose of 100 mg every 3 weeks, were alive after a median follow-up time of 21.5 months; four patients achieved complete remission of ATC that was confirmed by imaging analysis and clinical course [3]. Docetaxel arrests cell division by inhibiting microtubular depolymerization [14]. It has been reported that docetaxel increased the cell fraction in the G2/M phase of the cell cycle, considered to be the most radiosensitive phase, leading to radiation-sensitizing effects [15]. This may explain the mechanism underlying the synergistic effect of docetaxel and radiation. Taking these findings into account, we chose this combination therapy for the present case. Considering the age of the patient, the dosage of docetaxel was reduced and the dosing interval was extended. Although the dosage and the dosing interval of docetaxel were modified, ATC was well controlled during the whole clinical course. In addition, it should be noted that none of the severe adverse effects, with the exception of mild radiation dermatitis, was found at any time. Histopathological analysis during autopsy revealed complete remission of ATC. Our case appears to be the first reported in the literature involving complete remission of ATC after combination therapy, which could be pathologically confirmed. Our case report demonstrated the efficacy and a safety of combined therapy of radiotherapy with docetaxel to the aged patient with ATC. Although ATC is heterogeneous and it is difficult to standardize approach to treatment especially at an advanced stage, the best treatment involving a multimodal approach using a combination of radiotherapy and chemotherapy with docetaxel should be considered. Further analysis including the prospective randomized controlled study is required.
